# Tumor-exosomal miR-205-5p as a diagnostic biomarker for colorectal cancer

**DOI:** 10.1007/s12094-024-03647-6

**Published:** 2024-08-12

**Authors:** Yajing Zhao, Yapeng Zhao, Lisheng Liu, Guanghao Li, Yawen Wu, Yanan Cui, Li Xie

**Affiliations:** 1 Department of Clinical Laboratory, Shandong Cancer Hospital and Institute, Shandong First Medical University and Shandong Academy of Medical Sciences, Jinan, Shandong China; 2 Shandong Provincial Key Laboratory of Radiation Oncology, Cancer Research Center, Shandong Cancer Hospital and Institute, Shandong First Medical University and Shandong Academy of Medical Sciences, Jinan, Shandong 250117 Shandong Province China; 3 Department of Stomatology, Qinghai Red Cross Hospital, Xining, Qinghai China; 4 Shandong Second Medical University, Weifang, Shandong China

**Keywords:** Exosomes, Colorectal cancer, miR-205-5p, Diagnosis

## Abstract

**Background:**

Tumor-derived exosomal miRNAs play crucial roles in cancer diagnosis. Current studies aim to identify exosomal miRNAs associated with colorectal cancer (CRC) that are noninvasive, sensitive, and specific.

**Patients and methods:**

Exosomes were extracted from CRC patients and healthy donors via ultracentrifugation, followed by verification via transmission electron microscopy (TEM), qNano, and Western blot analysis. The differential expression levels and clinical characteristics of miR-205-5p were analyzed in CRC via data from The Cancer Genome Atlas (TCGA). Real-time quantitative PCR was used to assess the expression levels of exosomal miRNAs in 157 primary CRC patients, 20 patients with benign diseases, and 135 healthy donors. Predictions regarding target genes were made to guide further exploration of the disease’s etiopathogenesis through bioinformatics.

**Results:**

Compared with that in healthy donors, the expression of miR-205-5p in colorectal cancer (CRC) patients was significantly lower, as determined through analysis of the TCGA database. We conducted a prediction and analysis of the functional enrichment of downstream target genes regulated by miR-205-5p. A lower level of exosomal miR-205-5p in the serum of CRC patients than in that of healthy controls (*p* < 0.0001) and patients with benign disease (*p* < 0.0001) was observed. Furthermore, the expression levels of exosomal miR-205-5p were significantly lower in early-stage CRC patients than in the comparison groups (*p<0.001 and p* < 0.0001). Notably, the expression levels of exosomal miR-205-5p significantly increased postoperatively (*p* = 0.0053).

**Conclusions:**

The present study demonstrated that serum exosomal miR-205-5p may be a diagnostic biomarker for CRC.

## Introduction

Colorectal cancer (CRC) holds the position as the third most prevalent form of cancer, posing a significant risk to health. Around 40% of individuals diagnosed with CRC undergo relapse and recurrent metastasis, with a small fraction surviving beyond five years [1, 2]. Thus, the creation of efficient and accessible screening methods is vital for the prompt identification and correct staging of CRC patients. Such methods may include various types of fluid biopsies, such as proteins, tumor markers, circulating tumor cells [3], cell-free DNA [4], and exosomes [5], enabling early detection and accurate classification of CRC stages [6]. Exosomes, also referred to as extracellular vesicles, are secreted organelles with a single membrane and are 30 to 200 nm in diameter [7]. Cells communicate via exosomes [8]. Therefore, as essential messengers between cells, exosomes transmit signals to neighboring cells or distant anatomical sites by carrying cytokines [9], modify protein and genetic expression in receiving cells, and thus influence the functions of receiving cells. Exosomes play multiple roles in pathogenesis [10], metastasis [11] and immunity [12]. Exosomes include a variety of proteins [13], miRNAs [14] and DNA [15], which are apt to be transferred to target cells. For example, extensive deregulation of miRNAs has been shown in human cancers, which highlights their key role in the onset, development and metastasis of these tumors; hence, miRNAs are potential biomarkers for cancer.

MicroRNAs (miRNAs) are tiny, non-coding RNAs that are generally 20–25 nucleotide long [16] and unite with the target mRNA sequence after they enter the receptor cell [17]. Many studies have demonstrated that exosomal miRNAs are capable of mediating communication between cells and are thus involved in tumor carcinogenesis, metastasis, immunosuppression, angiogenesis, and other processes [18, 19]. Emerging evidence suggests that five types of exosomal miRNAs, namely miR-205, miR-19a, miR-19b, miR-30b, and miR-20a, are promising diagnostic markers of squamous cell lung cancer because of their decreased levels of circulation after resection of the lesion [20]. The level of serum exosomal miR-205 is increased in ovarian cancer tissues, and its overexpression is linked to cancer metastasis in ovarian carcinoma patients [11]. The relative expression of exosomal miR-1246 in serum is significantly greater in gastric cancer patients than in healthy controls [21]. Nasopharyngeal carcinoma cells discharge exosomes that contain certain types of miRNAs that play an inhibitory role in *T* cell proliferation, targeting the MAPK-1 and STAT pathways [22].

This study aimed to verify the different expression levels of serum exosomal miRNAs in CRC patients and in different groups by means of small RNA sequencing and fluorescence quantitative PCR. Consequently, exosomal miR-205-5p was chosen, and its diagnostic efficiency and clinical features were analyzed. Therefore, exosomal miR-205-5p is a novel diagnostic biomarker.

## Materials and methods

### Patients and clinical samples

In this study, 157 CRC patients, 135 healthy donors, and 20 patients with benign diseases hospitalized at Shandong Cancer Hospital from September 2017 to July 2018 were selected. Written consent was obtained from all participants. Tumor staging was estimated according to the AJCC Cancer Staging Handbook, 2017. The protocol received approval from the Shandong Cancer Hospital Affiliated with Shandong First Medical University and the Shandong Academy of Medical Sciences committee. Written informed consent was provided by all subjects in accordance with the Declaration of Helsinki. No patient had received antitumor treatment before serum collection, nor had any patient been diagnosed with endocrine, immune, or metabolic diseases. Serum collection was conducted on 17 out of 157 CRC patients who received surgical treatment after two months. Clinical features and diabetic histories of the patients were investigated (Table 1).

**Table 1:** Characteristics of CRC patients for differentially expressed exosomal miR-205-5p

		miR-205-5p	
Characteristic	No. cases	Median with interquartile range	*P*-value
Age(year)			
< 61	78	4.4729(4.0255–4.9203)	0.954
≥ 61	79	4.4001(3.9076–4.8925)	
Gender			
Male	105	4.6445(4.2411–5.0479)	0.094
Female	52	4.0158(3.4443–4.5872)	
Drinking status			
Yes	28	4.0204(3.0903–4.9504)	0.181
No	129	4.5265(4.1756–4.8774	
Diabetes status			
Yes	19	4.3734(3.2583–5.4885)	0.951
No	138	4.4449(4.0973–4.7924)	
Tumor position			
Rectum	92	4.4328(3.9982–4.8674)	0.920
Colon	65	4.4411(3.9231–4.9591)	
Tumor size			
< 30	50	4.5427(3.9112–5.1742)	0.748
≥ 30	60	4.3591(3.8134–4.9048)	
Unknown	47		
Lymph node metastasis status			
Yes	62	4.3886(3.9132–4.8640)	0.405
No	72	4.6332 (4.1118–5.1546)	
Unknown	23		
Distant metastasis			
Yes	35	4.1779(3.4966–4.8591)	0.386
No	122	4.5104(4.1304–4.8903)	

## Isolation of exosomes

The serum samples were subjected to ultracentrifugation at 10,000 × *g* for 30 min at 4 °C for the purpose of removing the cellular debris and then at 100,000 × *g* for 2 h at 4 °C for exosome precipitation [23]. The samples were subsequently analyzed via the following techniques.

## TEM assay

Transmission electron microscopy (TEM) was used to identify the purified exosomes. The exosome pellets were placed on grids coated with a 50-µL drop of 1% glutaraldehyde. After standing for 5 min, the grids were washed with a 100-µL drop of distilled water. After another standing time of 2 min, a 50-µL drop of uranyl-oxalate solution (pH = 7) was applied to the grids for 5 min and covered according to the instructions. Afterward, the grids were washed with distilled water (2 min each) and examined with a transmission electron microscope.

## Tunable resistive pulse sensing (TRPS)

The measurement of nanoparticle size was conducted via the TRPS technique and on a qNano (Izon Science Ltd.). Data analysis was conducted through the Izon Control Suite v.3.3.2.2000 (ibid).

## Immunoblotting

After being resolved by SDS-PAGE, equal amounts of exosomal proteins were transferred to PVDF membranes, which were then treated with a solution of 5% milk in Tris-buffered saline containing 0.1% Tween 20 for 1 h at 4 °C in dark conditions with rabbit primary antibodies against CD81, tumor susceptibility gene 101 (TSG101), and Golgi matrix protein 130 (GM130). Subsequently, they were incubated with HRP-conjugated secondary antibodies for 1 h at room temperature. Finally, the membranes were treated with ECL blotting detection reagents.

## Target genes and pathways

To elucidate the biological functions and potential molecular mechanisms associated with miR-205-5p in CRC, we conducted a pathway enrichment analysis. Initially, we utilized the LinkedOmics online tool (https://portal.gdc.cancer.gov) to identify mRNAs that were negatively correlated with miR-205-5p expression in the TCGA colorectal cancer cohort (TCGA-COADREAD). Genes with a correlation coefficient of less than −0.2 and a *p* value of less than 0.05 were deemed significant. Transcriptome data from the TCGA-COADREAD cohort in the TCGA database (portal.gdc.cancer.gov) were subsequently obtained. The R package ‘Limma’ was employed to perform differential expression analysis between the para-cancer and tumor groups. Genes meeting the criteria of logFC > 1 and *p* < 0.05 were identified as differentially expressed. Additionally, the TargetScan database (www.targetscan.org), the miRDB database (https://mirdb.org/mirdb/expression.html), and miRWalk (http://mirwalk.umm.uni-heidelberg.de/) were utilized to predict mRNAs associated with miR-205-5p. The intersection of the results from these three databases revealed molecules with a potential regulatory relationship with miR-205-5p. The R software package clusterProfiler was then employed to conduct Gene Ontology (GO) and Kyoto Encyclopedia of Genes and Genomes (KEGG) enrichment analyses of these intersecting genes. Finally, to explore the correlation between miR-205-5p and classic oncogenic pathways, the Hallmark gene set from MSigDB (https://www.gsea-msigdb.org/gsea/login.jsp) was obtained, and the gene set enrichment analysis (GSEA) method was utilized for enrichment analysis.

## RNA isolation and real-time PCR

TRIzol reagent was selected to extract aggregate RNAs, which were then reverse-transcribed into complementary DNA (cDNA) with the Mix-X miRNA First-Strand Synthesis Kit. Real-time PCR was conducted using TB-Green Premix Ex Taq II Reagent. U6 was used as an internal control [24]. All these procedures were performed in accordance with the specific manufacturer’s instructions. For each sample, analysis was performed in duplicate. In addition, the PCR was evaluated by melting curves, and the relative quantification of miRNA expression was performed via the ΔCT method (CtmiRNA-CtU6), as described in the previous Sect. [25].

## Statistical analysis

For the statistical analysis, SPSS 22.0 and GraphPad Prism 6.0 were used. Mann–Whitney U tests or t tests were used for comparisons, and paired t tests were performed to compare paired values. For multiple comparisons, one-way ANOVA was performed. The corresponding cutoff points were determined through ROC curves, where a *p* value < 0.05 was considered to indicate statistical significance.

## Results

### Characterization of isolated exosomes

**Fig. 1:**
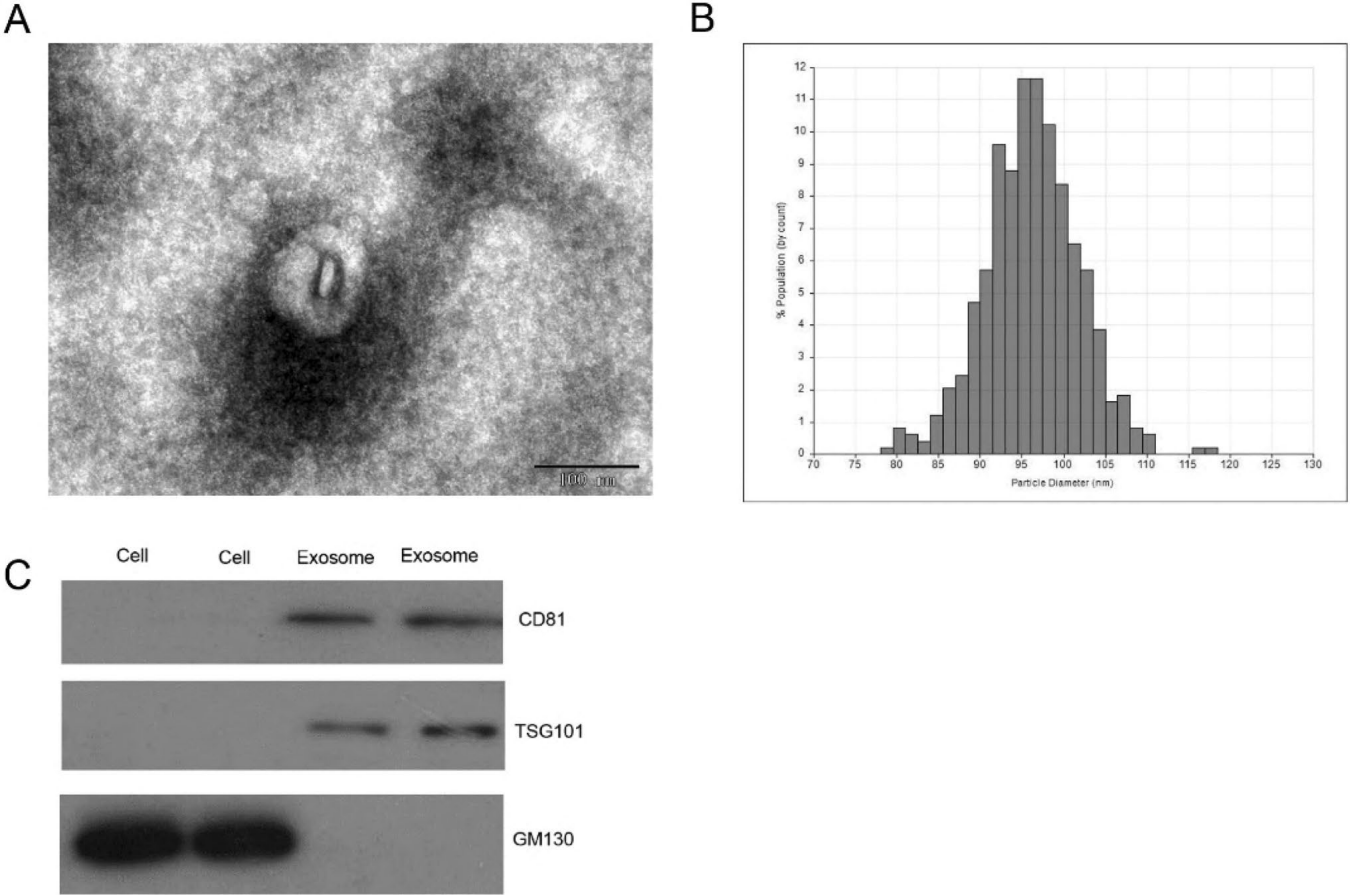
Characterization of isolated exosomes. (A) TEM images showing that the exosomes extracted from CRC patients was double-layer round vesicles with a diameter of 50–150 nm. (B) The diameter of the exosomes ranging from 50 to 150 nm was measured using qNano system. (C) Exosomal proteins CD81, TSG101 and GM130, a negative marker of the exosome was verified by immunoblotting

Serum exosomes from healthy donors and CRC patients were identified via TEM, qNano, and immunoblotting. The obtained microvesicles were cup-shaped and under 150 nm in diameter, which is consistent with the exosomal morphology observed via TEM (Fig. 1A). qNano analysis was employed to quantify the diameter of a single particle, which was determined to be a rounded particle with a diameter of 30–150 nm (Fig. 1B). As shown in Fig. 1C, TSG101 and CD81 were positive according to Western blotting and were enriched mainly in exosomes. GM130 is a matrix protein that regulates the structure of the Golgi apparatus (GA) and is generally employed as a negative control for exosomes [26], which are closely expressed in the cell. The above evidence revealed that the small vesicles obtained from the serum were exosomes because of their size and expression level of marker proteins.

### Differential expression of miR-205-5p in *pan*-*cancer* database

**Fig. 2:**
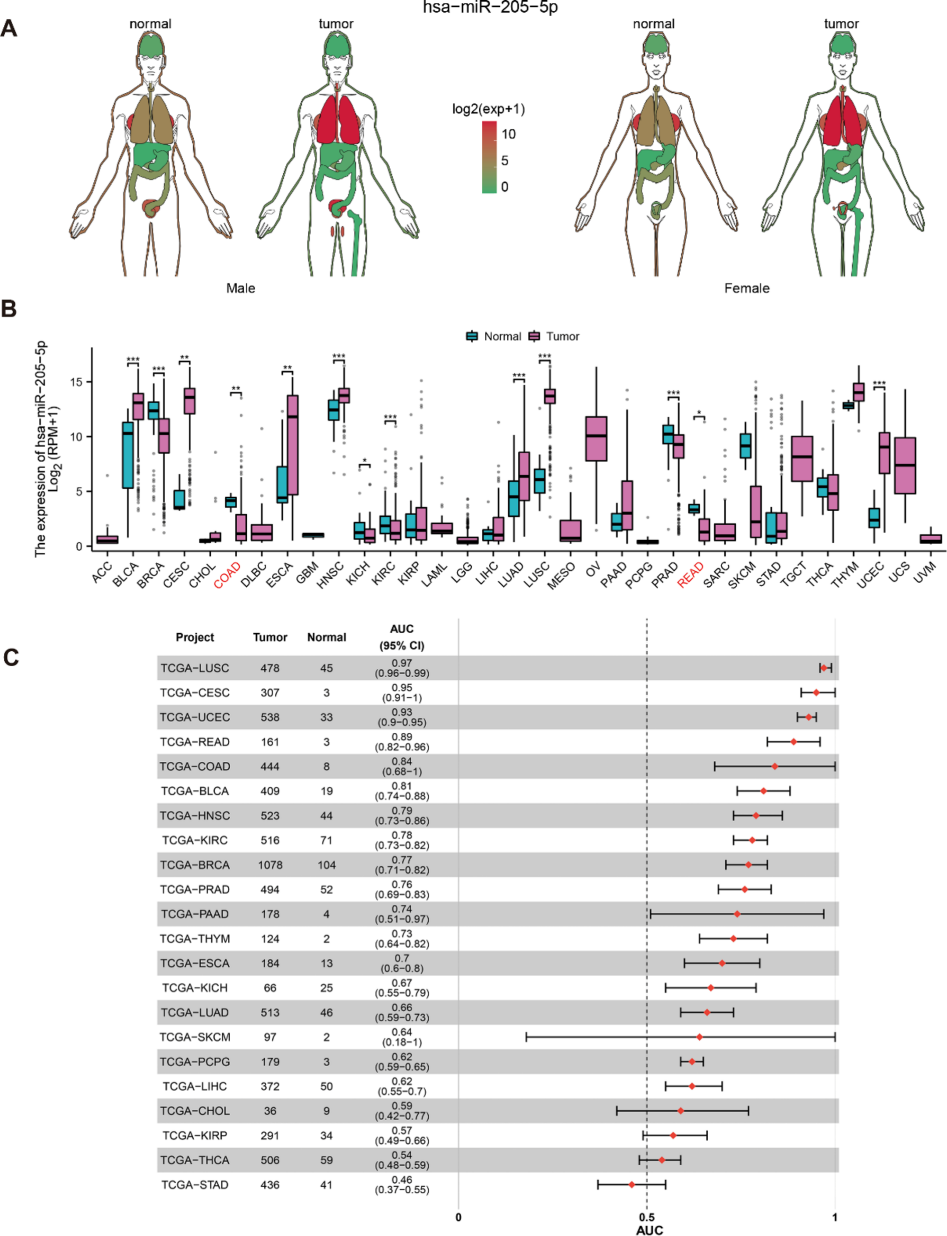
Differential expression of miR-205-5p in pan-cancer. (A) Distribution of miR-205-5p in the human body. (B) Differential expression of miR-205-5p in cancers of 33 types. (C) Area under the curve of miR-205-5p in different cancer types from TCGA database

To investigate the distribution of miR-205-5p in the human body, we analyzed data from the TCGA RNA-seq database. Our analysis revealed that miR-205-5p was located primarily in the lung, brain, stomach, and colorectum, and significant differences in its expression were detected between CRC patients and healthy donors (Fig. 2A). We subsequently performed a more in-depth analysis of miR-205-5p expression in various types of cancer by analyzing the TCGA RNA-seq data (Fig. 2B). Significant differences in the expression of miR-205-5p were observed across 33 types of cancer using paired or unpaired samples, excluding those lacking normal tissue data. miR-205-5p was significantly downregulated in BRCA (breast invasive carcinoma), COAD (colon adenocarcinoma), KICH (kidney chromophobe), KIRC (kidney renal clear cell carcinoma), PRAD (prostate adenocarcinoma), and READ (rectum adenocarcinoma) patients. Conversely, miR-205-5p expression was increased in BLCA (bladder urothelial carcinoma), CESC (cervical cancer), ESCA (esophageal carcinoma), HNSC (head and neck squamous cell carcinoma), LUAD (lung adenocarcinoma), LUSC (lung squamous cell carcinoma), and UCEC (uterine corpus endometrial carcinoma) patients. Finally, the diagnostic efficacy of miR-205-5p was analyzed, and the areas under the ROC curves (AUCs) of LUSC, CESC, UCEC, READ, COAD, and BLCA were 0.97, 0.95, 0.93, 0.89, 0.84, and 0.81, respectively (Fig. 2C).

### Identification of miR-205-5p in CRC

**Fig. 3:**
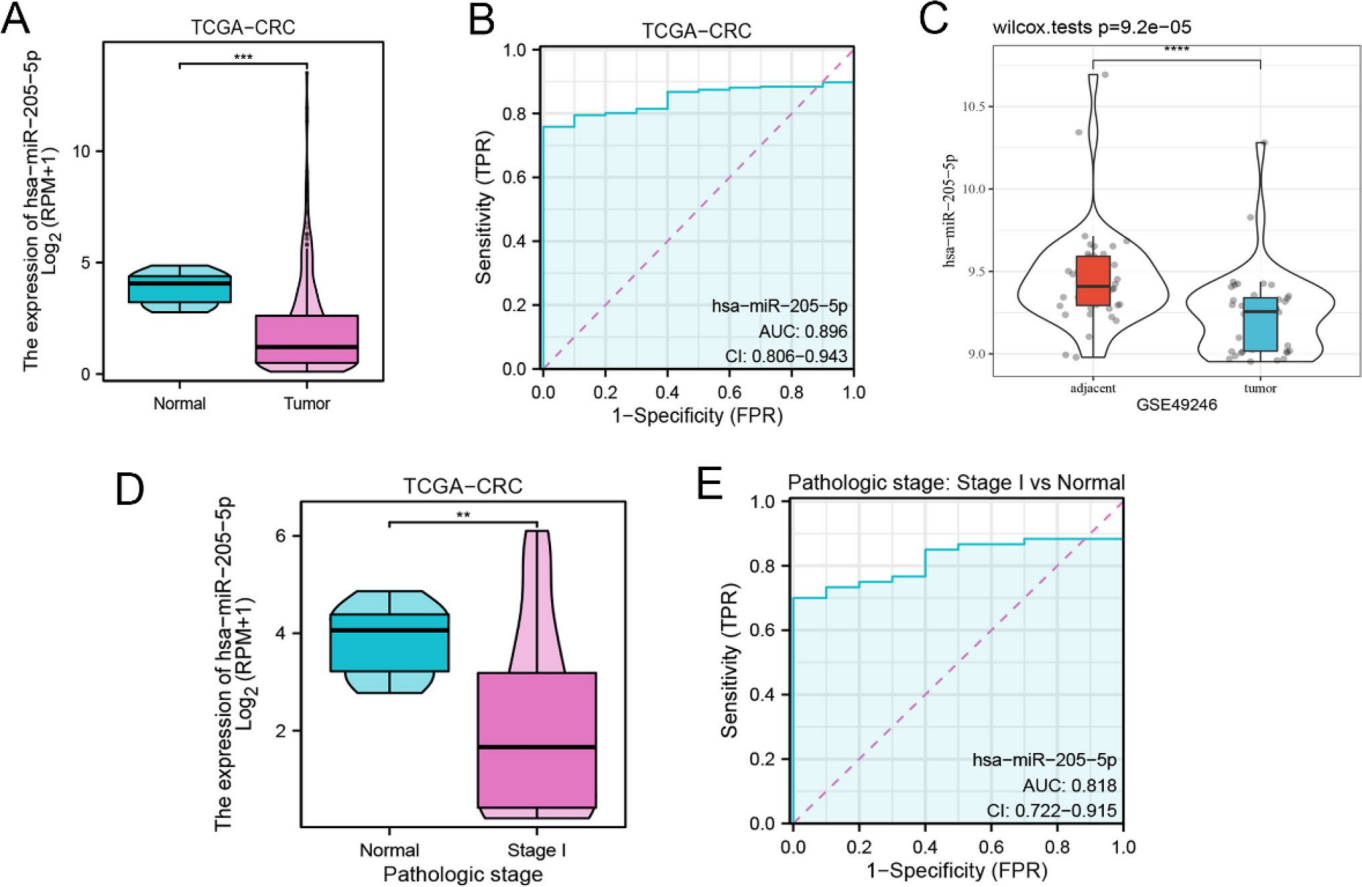
Identification of miR-205-5p in CRC. (A) The expression levels of miR-205-5p were notably higher in healthy donors as compared to CRC patients via TCGA database. (B) The AUC of miR-205-5p was 0.896 in CRC patients with comparison to healthy donors. (C) Mann–Whitney U test indicated that miR-205-5p was significantly decreased in CRC patients compare with healthy donors from GSE49246. (D) miR-205-5p expression levels in stageICRC were downregulated compared to healthy donors. (E) The AUC of miR-205-5p inIstage CRC patients was 0.818 compared to healthy donors

To verify the differential expression of miR-205-5p in CRC, we conducted an analysis of the TCGA database. The results revealed significant downregulation of miR-205-5p in CRC patients compared with healthy controls (Fig. 3A), as illustrated in Fig. 3B, and the AUC of miR-205-5p was 0.896. Adjacent tissues versus cancer tissues from the GSE49246 dataset revealed that the expression of miR-205-5p was markedly downregulated in cancer tissues (Fig. 3C).

To investigate whether miR-205-5p is diagnostic of early CRC via the TCGA database, we conducted a further analysis of the differential expression between stage I CRC patients and healthy donors (Fig. 3D), revealing a statistically significant difference. The AUC was calculated to be 0.818 (Fig. 3E).

**Fig. 4:**
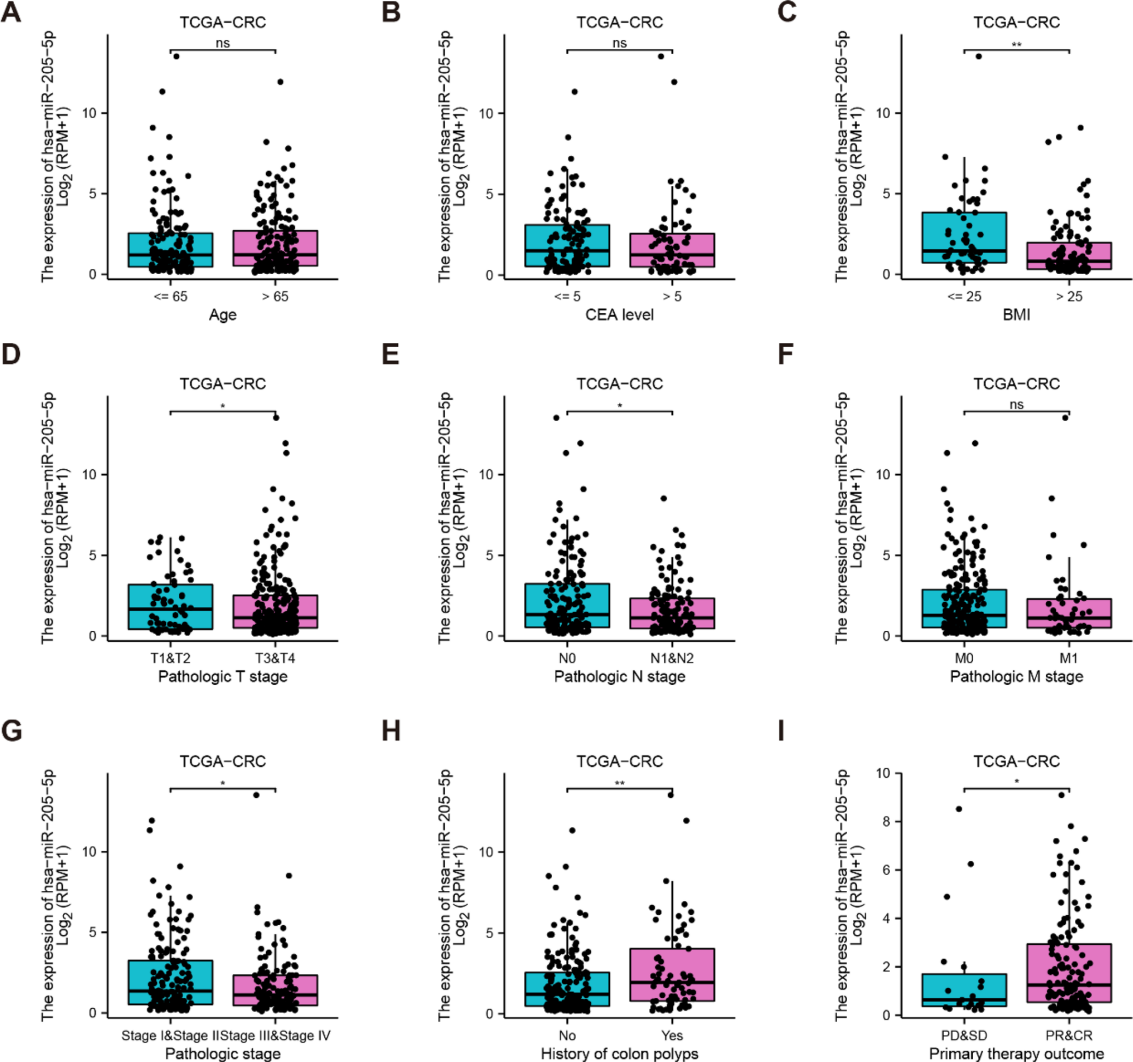
The correlation between miR-205-5p clinicopathological characteristics in CRC patients from TCGA database. (A) Age. (B) CEA. (C) BMI. (D) Pathological T stage. (E) Pathological N stage. (F) Pathological M stage. (G) Pathological stage (I+ II vs III + IV). (H) History of colon polyps. (I) Primary therapy outcome (PD + SD vs PR + CR)

An analysis of the relationships between miR-205-5p and clinical characteristics via TCGA data revealed that miR-205-5p was not correlated with age, CEA, or metastasis (Fig. 4A, 4B, and 4F). Nevertheless, there was significant variation when different BMI indices, pathologic T stages, and pathologic N stages were considered (Fig. 4C-E). Specifically, miR-205-5p displayed a significant difference in pathologic stage between I + II and III + IV (Fig. 4G). Furthermore, patients with colon polyps presented significantly higher expression levels of miR-205-5p than those without polyps (Fig. 4H). Additionally, lower expression of miR-205-5p was detected in patients with progressive disease (PD) and stable disease (SD) than in those with partial response (PR) and complete response (CR) (Fig. 4I). Considering the aforementioned findings, it can be inferred that miR-205-5p has the potential to serve as a biomarker for the early diagnosis and prognosis of CRC.

### Enrichment analysis of miR-205-5p in CRC

**Fig. 5:**
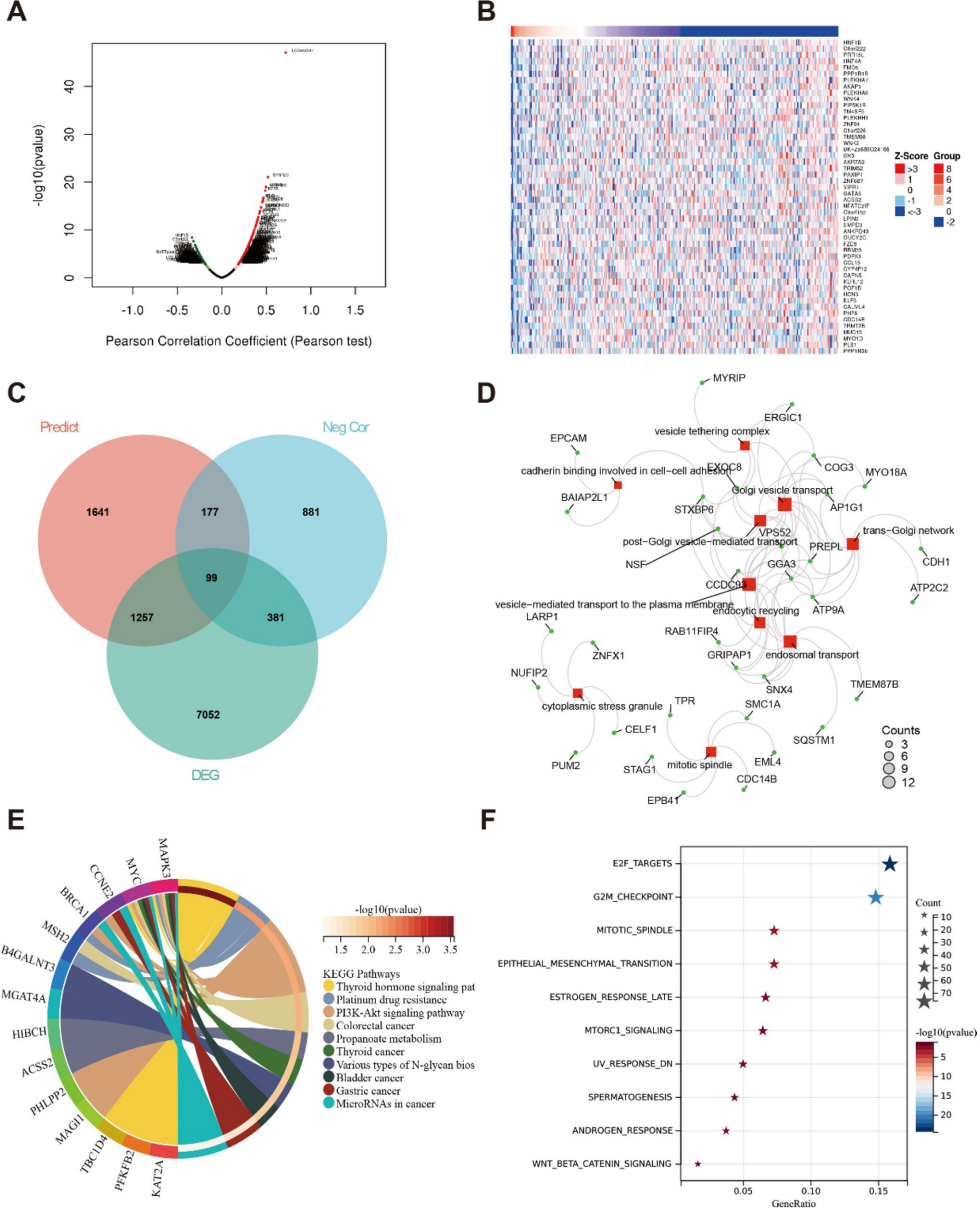
Functional enrichment analysis of miR-205-5p. (A) Correlation analysis of target genes with miR-205-5p, red dots represent positive correlation with miR-205-5p, Green dots represent negative correlation with miR-205-5p. (B) Heatmap illustrating the top 50 target genes negatively associated with miR-205-5p. (C) The Venn diagram illustrates the overlapping set of predicted target genes of miR-205-5p, target genes that exhibit a negative correlation with miR-205-5p and DEGs. (D) GO analysis. (E) KEGG pathway. (F) Hallmark pathway

It was predicted that miR-205-5p is involved in genetic and pathway regulation, providing a direction for exploring the pathogenesis of CRC through bioinformatics. To investigate the target genes of miR-205-5p, as shown in Fig. 5A, a correlation analysis of miR-205-5p with target genes from the TCGA CRC database was performed. A heatmap illustrating the top 50 target genes negatively associated with miR-205-5p is presented in Fig. 5B. The Venn diagram (Fig. 5C) illustrates the overlapping set of predicted target genes of miR-205-5p, target genes negatively correlated with miR-205-5p, and differentially expressed genes (DEGs) identified between healthy donors and CRC patients retrieved from the TCGA database. Gene Ontology (GO) analysis revealed significant enrichment of miR-205-5p in various cellular processes, specifically in Golgi vesicle transport, endosomal transport, vesicle tethering complex, vesicle-mediated transport to the plasma membrane, and cadherin binding involved in cell–cell adhesion (Fig. 5D). Additionally, miR-205-5p was predominantly enriched in microRNAs in cancer, colorectal cancer, the PI3K-AKT signaling pathway, the thyroid hormone signaling pathway, and platinum drug resistance (Fig. 5E). Importantly, we also conducted an analysis of the hallmark pathway, which exhibited notable enrichment in E2F targets, the G2M checkpoint, the mitotic spindle, and other related pathways (Fig. 5F). These findings indicate that miR-205-5p is associated with cancer onset and progression.

### Characterization of identified serum exosomal miR-205-5p

**Fig. 6:**
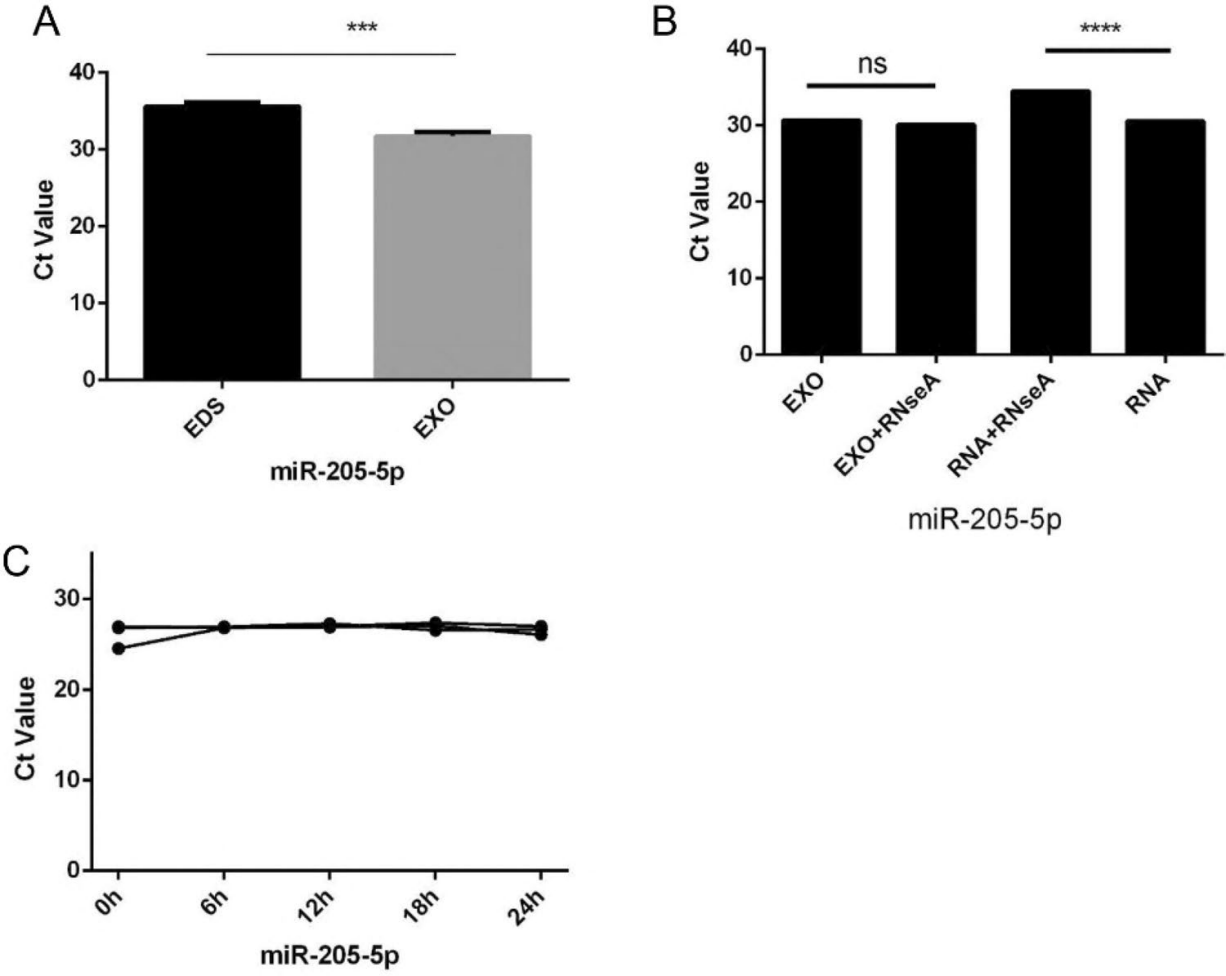
Characteristics of serum exosomal miR-205-5p. (A) Expression levels of miR-205-5p from EXO and EDS (B) Expression levels of miR-205-5p in exosomes treated with RNase A or RNA in isolation. (C) Expression levels of the miR-205-5p in exosomes incubated 0 h, 6 h, 12 h, 18 h,24 h at room temperature (***P < 0.001, ****P < 0.0001, ns, not significant)

The expression of miR-205-5p in the exosome-depleted supernatant (EDS) and exosomes was examined. Compared with that in EDS, miR-205-5p expression in exosomes substantially increased (Fig. 6A). Moreover, miR-205-5p expression in exosomes did not substantially change after RNase A treatment (Fig. 6B), which confirmed the stability of exosomal miR-205-5p. In simple words, these data demonstrated that miR-205-5p is expressed chiefly in exosomes and functions to protect miRNAs from being degraded by RNases. Notably, the level of miR-205-5p expression in the exosomes did not considerably change after exposure to room temperature for 0, 6, 12, 18, or 24 h (Fig. 6C).

### Exosomal miR-205-5p is associated with CRC

**Fig. 7:**
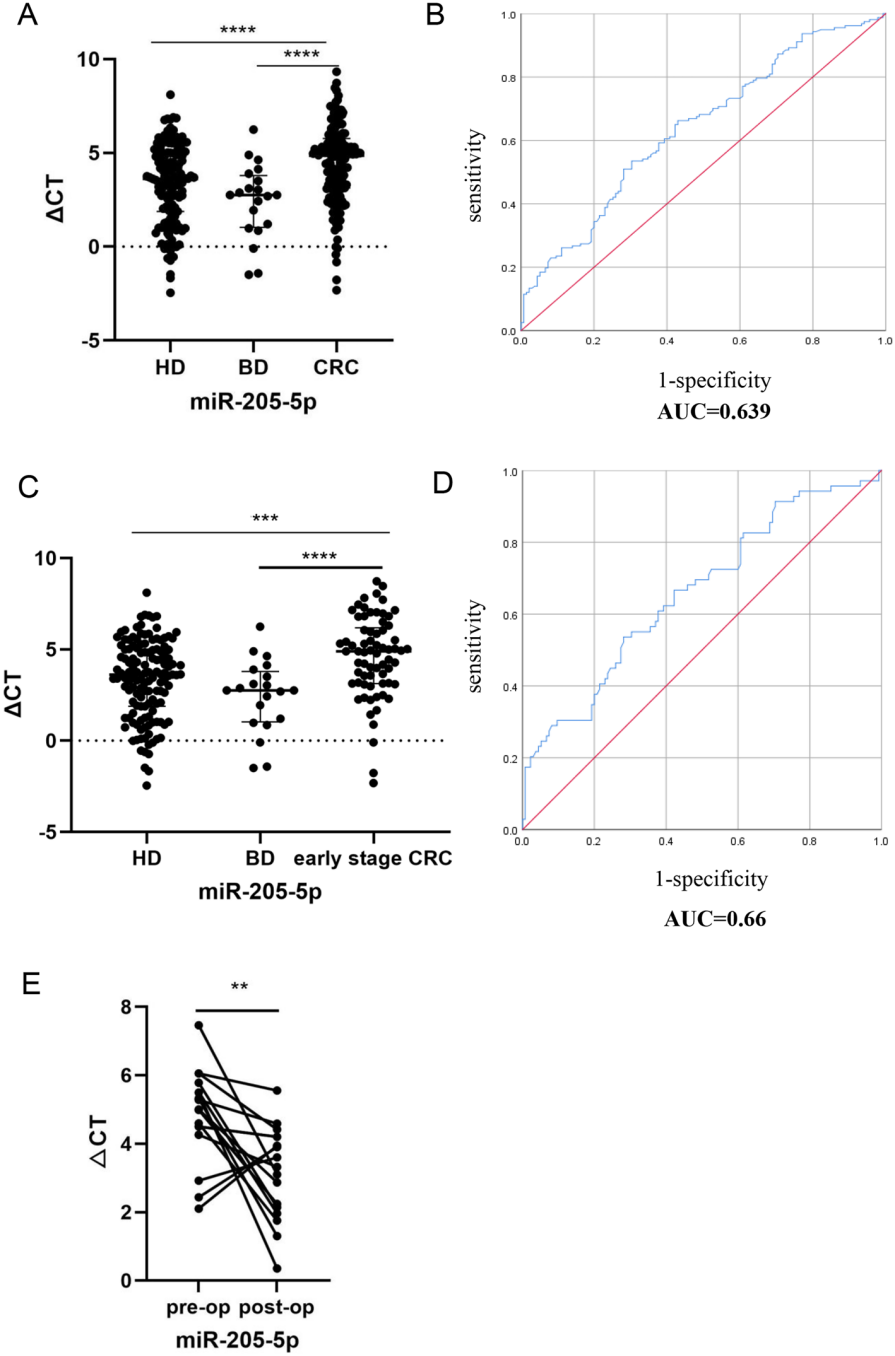
Exosomal miR-205-5p was downregulated in CRC patients. (A) Mann–Whitney *U* test showing that exosomal miR-205-5p decreased considerably in CRC patients in contrast with healthy donors and benign disease (***P < 0.001, ****P < 0.0001, ns, not significant). (B) The AUC of exosomal miR-205-5p for the detection of CRC was 0.639. (C) Mann–Whitney *U* test indicated that exosomal miR-205-5p was significantly decreased in early CRC patients (*I* = 16, *II* = 53) compare with healthy donors (*n* = 135) and benign disease (*n* = 20) (***P < 0.001, ****P < 0.0001, ns, not significant). (D) The AUC of exosomal miR-205-5p for the detection of early-stage CRC was 0.66.(F) The serum exosomal miR-205-5p levels was remarkably higher after operation than before operation (**p < 0.01)

To explore the potential value of exosomal miRNAs for cancer diagnosis, exosomal miR-205-5p expression levels were quantified in a total of 157 CRC patients, 135 healthy donors, and 20 patients with benign diseases via RT‒qPCR. The results in Fig. 7A suggest that the measured indicator in the serum decreases considerably in CRC patients (*p* < 0.0001, respectively) compared with healthy donors and those with benign disease (*p* < 0.0001, respectively) but is not as significant as the miRNAs detected in healthy subjects and patients with benign disease (*p* = 0.184, respectively).

Additionally, we investigated the correlation between the clinicopathological characteristics of patients with CRC and healthy donors with respect to miR-205-5p expression (Table 1). The outcomes suggested that they were not associated with age, sex, histology, or progression stage, among other factors.

In general, ideal clinical diagnostic biomarkers should have excellent sensitivity and specificity. In order to test whether serum exosomal miR-205-5p is an ideal diagnostic marker for CRC, ROC curve analysis was performed to determine its expression level. We found an area under the ROC curve (AUC) of 0.639 (95% CI: 0.576–0.702, *p* < 0.0001; Fig. 7B), and the sensitivity and specificity of detection were 53.5% and 69.2%, respectively.

Furthermore, the diagnostic efficacy was evaluated. As shown in Fig. 7C, early-stage CRC patients presented a lower level of exosomal miR-205-5p than did healthy donors and patients with benign disease (*p* < 0.001 and *p* < 0.0001, respectively). Next, ROC curves were generated to evaluate the performance of exosomal miR-205-5p in the early diagnosis of CRC. As shown in Fig. 7D, the AUC of miR-205-5p was 0.66 (95% CI: 0.58–0.739), and the sensitivity and specificity were 53.6% and 71.9%, respectively.

We continued to observe the expression of these molecules before and after surgical resection in 17 CRC patients. We found that the observed indicator substantially increased after the lesion was removed (*p* = 0.0053, Fig. 7E). The above findings suggest that tumor retention is likely to exert a negative effect on the observed indicator, making it a novel biomarker for monitoring surgical efficiency.

## Discussion

Colorectal cancer (CRC) is the most common malignant cancer worldwide and poses a major threat to people’s health and life [27]. Hence, controlling the progression of the disease at an early stage is highly important. Early diagnosis of CRC patients can result in a five-year survival of 90% [28]. However, delayed diagnosis occurs in nearly 60% of CRC patients, which is a survival rate of only 8–9%. Hence, a sensitive and specific biomarker is urgently needed for distinguishing patients with colorectal cancer. The results of this study indicated that exosomal miR-205-5p is considerably downregulated in CRC, suggesting that exosomal miR-205-5p is a potential biomarker for CRC diagnosis.

As previously reported, miR-205-5p is involved in the development of various types of cancers [29–31]. For example, the overexpression of miR-205-5p was detected in patients with non-small cell lung cancer compared with controls [32], and miR-205-5p was significantly downregulated in estrogen receptor-positive breast cancer by targeting NFIB [33]. Related studies have shown that miR-205-5p plays a regulatory role in epithelial‒mesenchymal transition by targeting PTEN via the PI3K/AKT signaling pathway in cisplatin-resistant nasopharyngeal carcinoma cells [34], and miR-205-5p inhibits PTK7, thereby affecting the proliferation, migration, and invasion of CRC[35]. Similarly, miR-205-5p is modulated by the lncRNA NEAT1 to promote CRC cell proliferation and migration through the regulation of the VEGFA signaling pathway [36]. In addition, an article reported that there was an anti-correlation between the expression level of miR-205-5p and the expression levels of BRCA1 and RAD17 targets in HNSCC [37]. Therefore, miR-205–59 might be used as a putative effective biomarker for CRC diagnosis.

This study confirmed that exosomal miR-205-5p might be used as a diagnostic biomarker for CRC. miR-205-5p was significantly downregulated in CRC patients compared with healthy controls via the TCGA database. Compared with that in control subjects, exosomal miR-205-5p was considerably downregulated in CRC patients. The AUC of exosomal miR-205-5p was 0.639, with a sensitivity of 53.6% and a specificity of 71.3%. The AUC of exosomal miR-205-5p was 0.66, with a sensitivity of 53.6% and a specificity of 71.9% in serum exosomal miR-205-5p from early-stage CRC patients. It is significantly increased after surgery, indicating that it is closely related to tumor occupation. Finally, miR-205-5p is expressed mainly in serum exosomes and is not affected by RNase A. In addition, the target genes of exosomal miR-205-5p and related pathways were predicted by bioinformatics, and we found that exosomal miR-205-5p was closely correlated with cancer.

However, the limitations of this study should be noted. First, the small cohort size did not render sufficient statistical support for the conclusion. In future studies, a larger sample size and longer-term clinical follow-up are expected. Second, we did not consider the diagnostic efficacy of exosomal miR-205-5p in combination with routine tumor biomarkers of CRC, such as CEA and CA199, since relative data concerning healthy donors were not collected. We intend to further explore the mechanism and prognosis of serum exosomal miR-205-5p.

In summary, this study revealed that the expression levels of exosomal miR-205-5p were markedly lower in CRC patients, including early-stage CRC patients. This finding is valuable for CRC diagnosis and is of practical significance for CRC treatment. Therefore, this study provides evidence that exosomal miR-205-5p is a promising diagnostic biomarker for CRC in clinical practice.

## Data Availability

The data that support the findings of this study are available from the corresponding author upon reasonable request.
